# The efficacy of internet-based cognitive behavior therapy for psychological health and quality of life among breast cancer patients: a systematic review and meta-analysis

**DOI:** 10.3389/fpsyg.2024.1488586

**Published:** 2025-01-08

**Authors:** Tang Lin, Yin Ping, Cai Ming Jing, Zhi Xiao Xu, Zhu Ping

**Affiliations:** ^1^Northern Jiangsu People's Hospital, Yangzhou University, Yangzhou, China; ^2^Jiangsu Cancer Hospital, Nanjing Medical University, Nanjing, Jiangsu, China

**Keywords:** internet, cognitive behavior therapy, mental health, quality of life, breast cancer, systematic review, meta-analysis

## Abstract

**Objective:**

To systematically investigate the effect of Internet-based cognitive-behavioral therapy (ICBT) on depression, anxiety, fatigue and quality of life (QOL) in patients with breast cancer (BC).

**Methods:**

A systematic review with meta-analysis and qualitative evidence synthesis was performed. Ten databases, including PubMed, Web of Science, EMBASE, the Cochrane Library, CINAHL, JBI Chinese Biomedical database (CBM), China National Knowledge Infrastructure (CNKI), VIP, and Wanfang, were searched from the start till August 2023. Published studies in English or Chinese were eligible for randomized and clinical controlled trials determining the efficacy of ICBT among BC survivors. The quality of the evidence was evaluated at the study level and the outcome level.

**Results:**

In total, 11 clinical trials on 1,307 patients with BC (ICBT: 671, control: 636) were systematically reviewed. We found that ICBT is effective in alleviating psychological distress in BC survivors, and the quality of all studies was moderate. The meta-analysis indicated that ICBT affected primary outcomes of anxiety (standardized mean difference [SMD] = −0.71, 95% confidence interval [CI]: −1.19, −0.24, *p* < 0.0001), depression (SMD = −0.67, 95% CI: −1.07, −0.27, *p* < 0.0001), fatigue (SMD = −1.23, 95% CI: −2.37, −0.08, *p* < 0.0001) and QOL (SMD = 0.79, 95% CI: 0.21, 1.37, *p* < 0.00001).

**Conclusion:**

This meta-analysis indicates that ICBT, as a type of psychosocial rehabilitation, can mitigate the incidence of depression, anxiety, and fatigue and improve the quality of life among patients with BC. Nevertheless, the effect is not distinct, and multi-centered randomized controlled trials with larger cohorts are warranted to verify the effectiveness of ICBT.

## Introduction

The latest GLOBOCAN 2020 cancer burden data reports that breast cancer (BC) has overtaken lung cancer as the most prevalent cancer globally. The number of new cases of BC is as high as 2.26 million, accounting for approximately 11.7% of the new cases of cancer (Sung et al., 2021). The average 5-year survival rate of patients with BC has been significantly prolonged by up to 90% with the advent of new therapeutic methods (Li Y. H. et al., 2023). The journey from diagnosis through treatment and beyond is often fraught with emotional distress, including depression, anxiety, and stress, which can profoundly impact the quality of life (QOL) of breast cancer patients (Karveli et al., 2023; Rodríguez-Gonzalez et al., 2023). Mental health is important for the success of BC recovery. Good mental health can empower patients to regain confidence and a positive attitude to deal with the challenges of recovery (Li Y. et al., 2023). Consequently, rehabilitation support focusing on improving the mental health of BC survivors is important.

To deal with the psychological burden, drug treatment has been used to treat the psychological symptoms of patients with BC, including antidepressants, anti-anxiety drugs, and psychotropic drugs, which have side effects (Gosain et al., 2020). Hence, non-pharmacological interventions for psychological problems are relatively less harmful. The World Health Organization (WHO) has mandated the inclusion of psychosocial support as a component of cancer care (Kohrt and Song, 2018). Presently, several non-pharmacological interventions have been reported, including peer education, narrative therapy, aerobic exercise, and cognitive behavior therapy. These interventions help in the development of non-drug therapeutic approaches for psychological issues and are effective in helping BC survivors alleviate their psychological symptoms (Toija et al., 2024; Zhang et al., 2024; Yao et al., 2024; Ye et al., 2018).

Among the aforementioned psychotherapy measures, cognitive behavioral therapy (CBT) is a valuable treatment modality for alleviating the distress of cancer survivors and possesses unique advantages (Getu et al., 2023). CBT is a structured, short-range, cognition-oriented psychotherapy, which largely focuses on mental disorders such as depression and anxiety, and psychological problems due to unreasonable cognition (Yang et al., 2018). CBT can be given as individual or group therapy based on face-to-face or online interventions (Zhao et al., 2023). Nevertheless, the implementation of CBT should be based on trained professional teams, with sufficient time and professional psychotherapists, ensuring that the patients = have access to CBT, so accessibility and scalability remain significant challenges.

The advent of digital technologies has introduced novel avenues for delivering psychological interventions, with Internet-based cognitive behavior therapy (ICBT) emerging as a particularly promising approach (Yu et al., 2023). A study reported that approximately 81% of BC survivors searched for online cancer information, and cancer survivors can use online resources to search for information, get support, and share their experiences (Ludwigson et al., 2023). ICBT leverages the ubiquity of the internet to provide accessible, flexible, and scalable psychological support, overcoming many of the logistical barriers associated with traditional therapy (Murphy et al., 2021). This modality allows patients to engage with therapeutic content at their own pace and convenience, making it particularly suitable for those with limited access to face-to-face care or those who may feel stigmatized seeking psychological help. Despite the growing interest and application of ICBT in various clinical populations, its efficacy in breast cancer patients remains a subject of considerable debate. While some studies have reported positive outcomes, others have raised concerns about the variability in intervention design, delivery, and patient adherence, which may influence treatment effectiveness. A systematic review and meta-analysis are therefore warranted to synthesize the existing evidence and provide a comprehensive assessment of ICBT’s impact on the psychological health and QOL of breast cancer patients. Hence, in this systematic review and meta-analysis, we aimed to evaluate the quality of evidence and determine the efficacy of the ICBT in reducing anxiety, depression, fatigue, and improving quality of life among breast cancer patients.

### Method

This study followed the Preferred Reporting Items for Systematic Reviews and Meta-Analyses (PRISMA) guidelines (Shamseer, et al., 2015). The protocol for this systematic review is registered on PROSPERO (CRD42023468838).

### Eligibility criteria

Full-text articles designed for clinical trials that fulfilled the criteria for randomized clinical trials were included. The inclusion criteria for articles were as follows: P: Patients with a confirmed diagnosis of BC should be more than 16 years old. There were no limits based on gender, Cancer stage, sex, or ethnicity. I: Interventions should fulfill the definition of cognitive-behavioral therapy or should be used as a component of it. Internet-based instruments, such as websites, mobile, smartphone applications, or emails, should be used. C: To control external variables and obtain overall trends, we included studies in which rehabilitation interventions in experimental groups were compared with the following control programs: education, routine treatment, and offline intervention. O: Outcomes related to mental health and QOL, such as depression, anxiety and fatigue.

### Search strategy

According to the PRISMA guidelines, we systematically obtained data from inception to August 2023 for randomized controlled trials (RCTs) from the following ten databases: PubMed, Web of Science, OVID, EMBASE, the Cochrane Library, CINAHL, JBI Chinese biomedical database (CBM), China National Knowledge Infrastructure (CNKI), VIP, and Wanfang. Central searches were performed using the following terms: search filters were structured for 4 integrated search themes namely, “Breast cancer,” “Internet,” “Cognitive behavior therapy,” and “Randomized controlled trial” using the combination of medical subject heading terms. Search terms of each theme were combined with “OR,” and search themes were then combined with “AND.” The keywords were searched alone or in combination with other keywords such as “breast neoplasms” OR breast carcinoma” OR “breast cancer” OR “breast tumor” OR “mammary cancer” OR “malignant neoplasm of breast” AND “internet-based” OR “web-based” OR “digital technology” OR “online” OR “technology-based” OR “electronic” OR “mobile” OR “e-Health” OR “network” AND “cognitive behavioral therapy” OR “behavioral therapy” OR “cognitive therapy” AND “randomized controlled trial” OR “randomized clinical trial” OR “random.*.”

### Study selection

The review was performed and reported in accordance with the PRISMA guidelines. After removing duplicate studies, two reviewers independently screened the titles and abstracts of all relevant studies. The full-text articles were finally obtained and the articles based on the inclusion criteria were included.

### Quality appraisal

Two authors (TL and CMJ) separately determined the risk of bias based on the Cochrane Handbook (Cumpston et al., 2019) in terms of allocation sequence, blinding of participants, personnel, assessor, incomplete outcome data, and selective reporting bias. The risk of bias for each outcome was categorized into the following three levels: low, high, and unclear.

### Data extraction and analysis

Standardized extraction forms were used to obtain the data from the included articles. Data included sample characteristics, number of participants in experimental and control groups, duration of interventions, intervention methods, follow-ups, and outcomes. Two reviewers independently performed data extraction from each study. Furthermore, any inconsistencies were resolved through discussion with a third reviewer. Authors of the studies were contacted if more data were required. The Review Manager 5.3 software was used for data analysis. Furthermore, all numeric outcome data were added twice to prevent data entry errors. Heterogeneity was quantified using *I^2^* statistics. A fixed effects model was used for *I^2^* < 50%. In the case where a significant heterogeneity (*I^2^* ≥ 50%) was observed, a random effects model was used. For continuous data that used the same scale, the mean difference (MD) was selected as the summary measure. The standardized mean difference (SMD) was used when the same outcome was measured by different scales. The SMDS was 0.20, 0.50, and 0.80, which were divided into small, medium, and large effect sizes, respectively (Hedges and Pigott, 2001). Outcomes were collected and presented in a narrative form and tables.

## Results

### Search process

**Figure 1:**
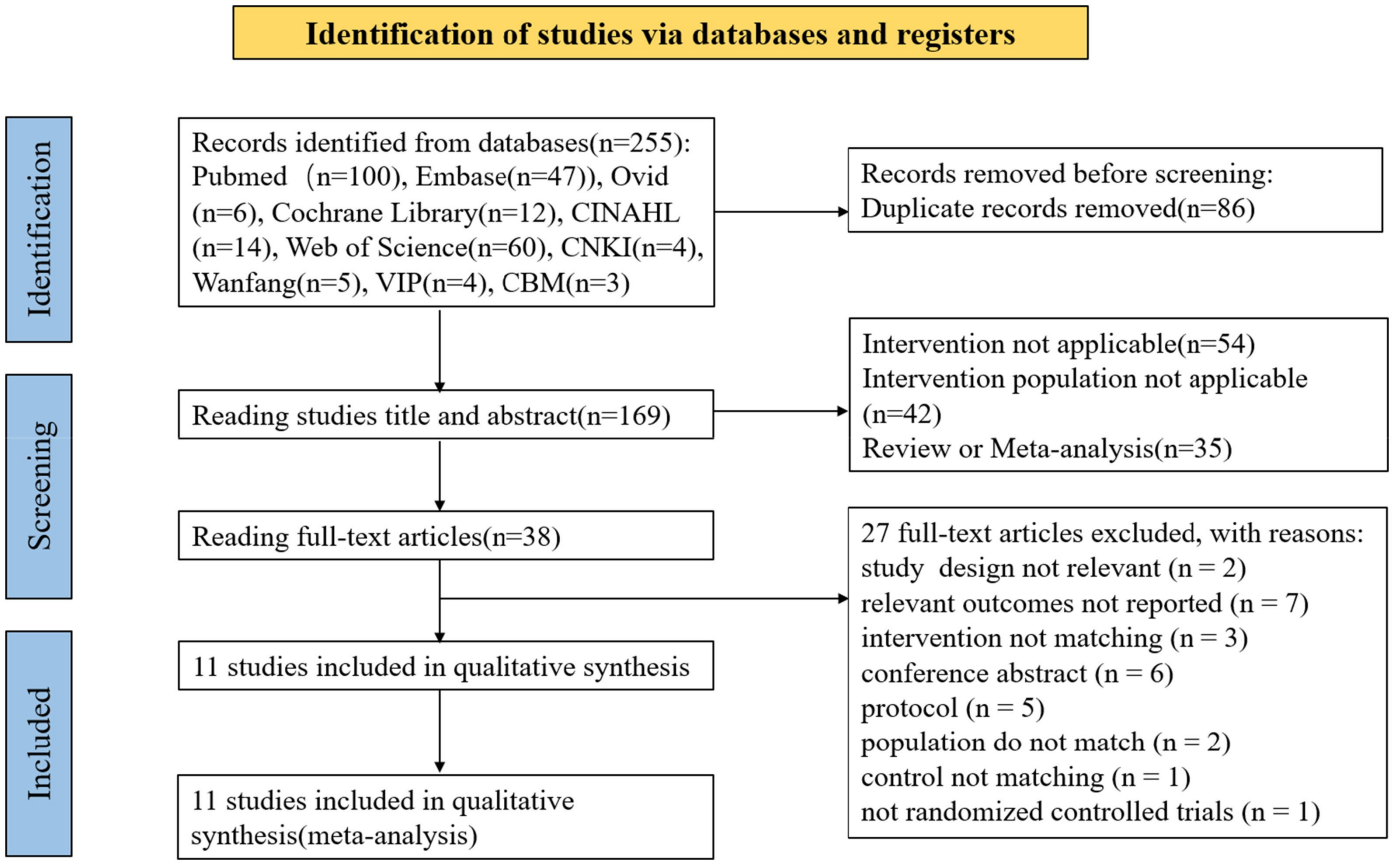
Flowchart depicting the literature search and study selection.

The selection process is presented as a flow chart in Figure 1. We obtained 255 articles from the ten databases at the beginning of the study. Due to the presence of duplicate articles, 86 documents were excluded, and 169 articles were included and screened for further assessment. After screening the titles and abstracts according to the inclusion criteria, the number of references was reduced to 35. Lastly,11 trials including 1,307 patients with cancer, were included in this study (Yu et al., 2022; Gong et al., 2021; Chen, 2022; Holtdirk et al., 2021; Rosen et al., 2018; Akkol-Solakoglu and Hevey, 2023; Abrahams et al., 2017; Peng et al., 2022; Wang et al., 2022; Amidi et al., 2022; van den Berg et al., 2015). In the title/abstract and full-text screening stages, kappa coefficients were 92.9% (*p* < 0.001) and 81.3% (*p* < 0.001), respectively, indicating that all reviewers had the same view of the literature.

### Characteristics of the included studies and participants

The characteristics of the included 11 trials are summarized in Table 1. We included RCTs published from inception till August 2023. Five studies were performed in China, whereas others were performed in Netherlands (*n* = 2), Germany (*n* = 1), the United States (*n* = 1), Denmark (*n* = 1), and Ireland (*n* = 1). We included 11 RCTs with 1,307 participants. Of the 11 trials included, nine explicitly stated that the breast cancer patients included were women (*n* = 9), while the remaining two studies did not (*n* = 2). The mean age of all included patients was 34.57 to 63.13 years. The sample size ranged from 40 to 363, with an average of 119. Most patients with BC had completed their initial cancer treatment, surgery, or radiation and chemotherapy, and many of them were diagnosed for less than 5 years.

**Table 1:** Characteristics of included studies for review (*N* = 11).

Author (year)	Sample	Mean age (± SD)	Cancer stage	Current treatment	Intervention group: form of intervention	Control group: intervention	Outcome
		Gender			Duration, frequency, length of program		
Yu et al. (2022)	IG20 CG20	IG47.1 ± 4.38 CG46.45 ± 3.47 Female	I-III	Surgery	networked group brief behavioral treatment Unclear, 1time/week, 8 weeks	Health education classes	GAD-7,PHQ-9,PSQI,FACT-B
Gong et al. (2021)	IG37 CG37	IG45.24 ± 5.04 CG47.03 ± 5.84 Female	I-II	Perioperative period	computerized cognitive behavioral therapy 20 ~ 30 min, Five stages of perioperative period	Health education classes	SAI, PHQ-9, AIS
Chen, 2022	IG35 CG38	IG42.91 ± 8.34 CG44.63 ± 10.43 Not reported	I-III	Surgery/chemotherapy/antiestrogen therapy/radiation treatment	networked group brief behavioral treatment 90 min, 1time/week, 6 weeks	Usual care	ISI, PSQI, SAS, SDS
Holtdirk et al. (2021)	IG181 CG182	IG50.07 ± 8.51 CG49.8 ± 7.98 Female	Not reported	Post-treatment	holistic Internet-based intervention Optimune Unclear	Usual care	WHO QOL BREF, IPAQ, ISI, BFI, PA-F12, IES-R, PHQ-9
Rosen et al. (2018)	IG57 CG55	IG51.40 ± 10.73 CG53.22 ± 9.91 Female	0-IV	Surgery/chemotherapy/antiestrogen therapy/radiation treatment	Mobile App-Delivered Mindfulness Training Unclear, 6-month AMT subscription, 10-day foundation course	Waiting list	FACT-B, MAAS
Akkol-Solakoglu and Hevey (2023)	IG49 CG23	IG47.12 ± 7.92 CG49.30 ± 9.66 Female	0-IV	Post-treatment	Internet-delivered cognitive behavioral therapy Unclear, 8 weeks	Health education classes	HADS, EORTC-QLQ, CWS, MOS-SSS
Abrahams et al. (2017)	IG66 CG66	IG52.5 ± 8.2 CG50.5 ± 7.6 Female	I-III	Surgery/chemotherapy/antiestrogen therapy/radiation treatment	Internet-Based Cognitive Behavioral Therapy Unclear, 3 face-to-face sessions and a maximum of 8 Web-based modules.	Waiting list	CIS, SIP-8, BSI-18, EORTC-QLQ
Peng et al. (2022)	IG28 CG29	Not reported Female	I-IV	mastectomy/conservative therapy/breast construction	Internet-Based mindfulness-based cancer recovery 1.5 h, 1time/week, 6 weeks	Usual care	FCRI-SF, EFMQ, EORTC-QLQ
Wang et al. (2022)	IG51 CG52	IG45.37 ± 7.59 CG48.17 ± 8.05 Not reported	0-IV	Surgery/chemotherapy/antiestrogen therapy/radiation treatment	Internet-Delivered Mindfulness-Based Cancer Recovery Intervention > 30 min, daily, 4 weeks	Usual care	MDASI-C, FACT-B
Amidi et al. (2022)	IG77 CG54	IG53.5 ± 8.9 CG54.0 ± 7.8 Female	I-III	Chemotherapy/Radiotherapy/Antihormone therapy	Internet-delivered cognitive-behavioral therapy Unclear, 6 weeks	Waiting list	rMEQ, PSQI, ISI, FACIT-F, BDI-II
van den Berg et al. (2015)	IG70 CG80	IG51.44 ± 8.30 CG50.18 ± 9.15 Female	Not reported	Surgery/chemotherapy/antiestrogen therapy/radiation treatment	Web-Based Self-Management for Psychological Adjustment Unclear, 16times/week, 2-4 months	Usual care	SCL-90, ICQ, CEQ, CIS, CWS, CAS, EORTC QLQ-C30, HADS, IES, PAQ, RS12

IG, intervention group; CG, control group; N, number of participants; SD, standard deviation; GAD-7, Generalized Anxiety Disorder Scale; SAI, State Anxiety Inventory Scale; SAS, Self-Rating Anxiety Scale; HADS-A, Hospital Anxiety and Depression Scale-Anxiety; PHQ-9, The Patient Health Questionnaire-9; SDS, Self-rating depression scale; HADS-D, Hospital Anxiety and Depression Scale-Depression; BDI-II, Beck Depression Inventory, Second Edition; BFI, Brief Fatigue Inventory; CIS, The CIS-Fatigue Severity; FACIT-F, The Functional Assessment of Chronic Illness Therapy–Fatigue; AIS, Athens Insomnia Scale; ISI, Insomnia Severity Index; PSQI, Pittsburgh Sleep Quality Index. EORTC-QLQ, European Organization for Research and Treatment of Cancer Quality of Life Questionnaire Core 30; WHO QOL BREF, WHO quality of life questionnaire BREF; PA-F12, Fear of Progression Questionnaire; IES-R, Intrusion scale of the Impact of Event Scale-Revised, 7 items; MAAS, the 15-item Mindful Attention Awareness Scale; CWS, Breast Cancer Worry Scale; MOS-SSS, Medical Outcomes Study Social Support Survey; SIP-8, Sickness Impact Profile 8; BSI-18, Brief Symptom Inventory 18; FCRI=SF, The Short Form of the Fear of Cancer Recurrence Inventory; EFMQ, Five Facet Mindfulness Questionnaire; MDASI-C, The Chinese version of the MD Anderson Symptom Inventory; rMEQ, Reduced Morningness-Eveningness Questionnaire; SCL-90, Symptom Checklist-90 items; CEQ, Cancer Empowerment Questionnaire; ICQ, Illness Cognition Questionnaire; IES, Impact of Event Scale; PAQ, Positive Adjustment Questionnaire; RS12, Remoralization Scale-12.

### Characteristics of interventions

We did not find any uniform commonalities across all included studies. For the trials included in this meta-analysis, except for one study that used a mobile App for the intervention (Rosen et al., 2018), all experimental groups used ICBT. Interventions differed in terms of tools, duration, frequency, and settings. The implementers of ICBT included the mindfulness cognitive psychology team (*n* = 6) (Yu et al., 2022; Chen, 2022; Holtdirk et al., 2021; Rosen et al., 2018; Abrahams et al., 2017; van den Berg et al., 2015), licensed cognitive behavioral therapists (*n* = 2) (Wang et al., 2022; Amidi et al., 2022), the student therapists (*n* = 1) (Akkol-Solakoglu and Hevey, 2023), primary nurse (*n* = 1) (Gong et al., 2021), and one more group that is not mentioned (*n* = 1) (Peng et al., 2022)^.^ The range of study duration was 4 ~ 24 weeks with a median of 10 weeks. In this study, most included studies were performed using mixed modules, and the modules of each study were different. For instance, a fixed 16-week modular program for four phases of adjustment to breast cancer (namely looking back, emotional processing, strengthening, and looking ahead), included information, assignment, assessment, and video (van den Berg et al., 2015). A 4-week Imber program included the experience of mindfulness-connecting mind and body, power of awareness-emotion and thought, stress management and self-compassion, and incorporating mindfulness into daily life. Four of the included studies were published study protocols (Holtdirk et al., 2021; Akkol-Solakoglu and Hevey, 2023; Abrahams et al., 2017; van den Berg et al., 2015).

### Bias in the studies

**Figure 2:**
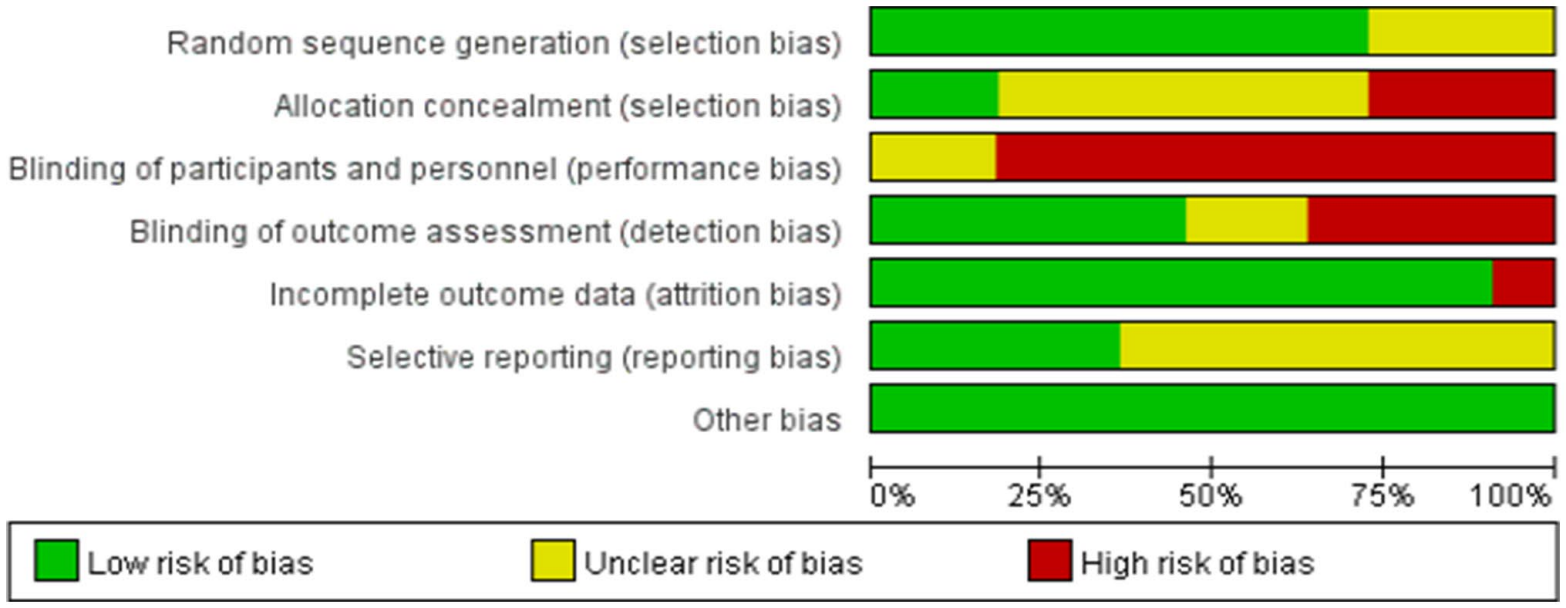
The risk of bias graph.

**Figure 3:**
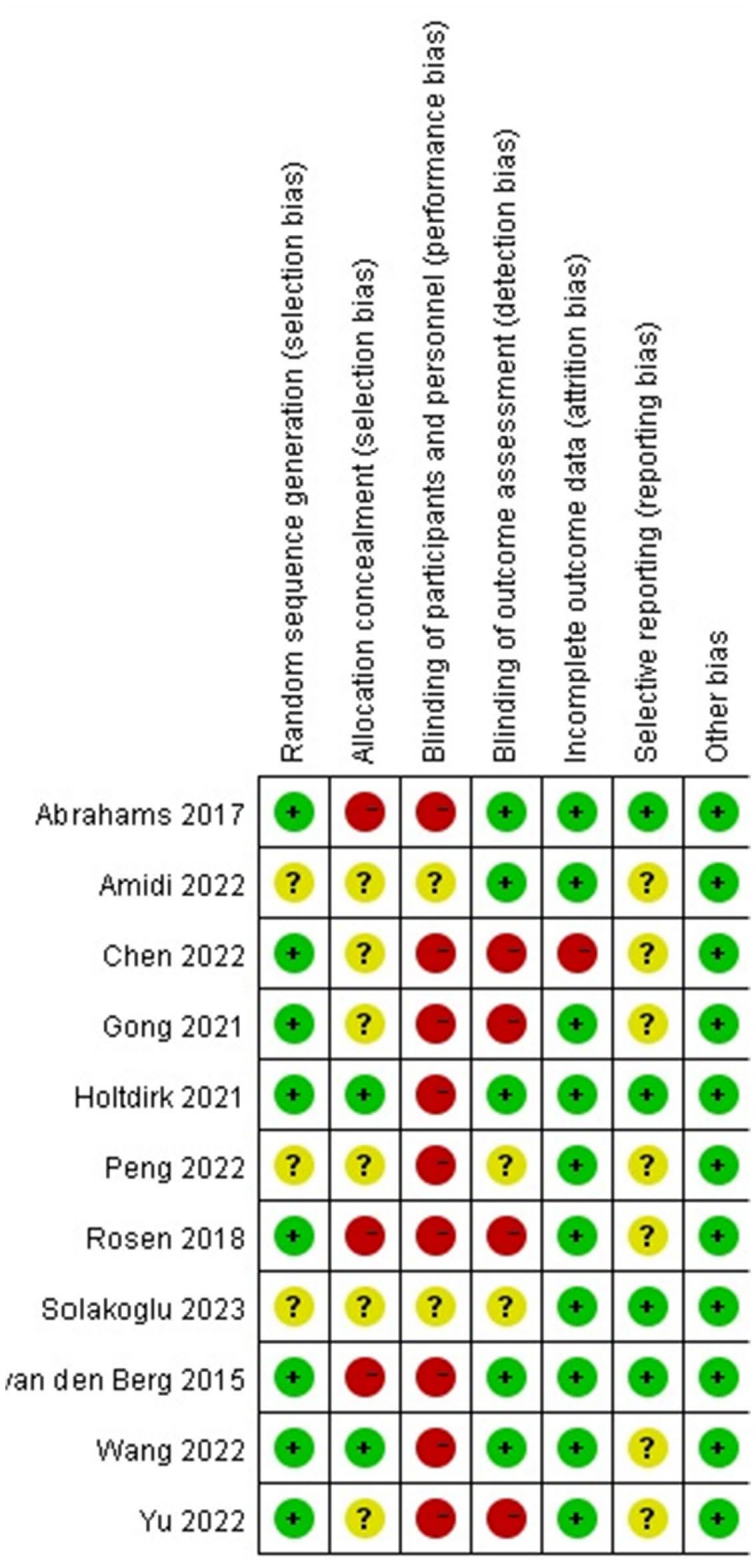
Risk of bias summary.

No RCT fulfilled all the requirements for a low risk of bias. Eight studies included appropriate sequence generation procedures, and only 2 studies used assigned hiding (Holtdirk et al., 2021; Wang et al., 2022). Due to the characteristics of the intervention, most studies (*n* = 9) did not blind participants and therapists. None of the studies were blind to researchers or patients, whereas five studies were blind to the evaluators (Holtdirk et al., 2021; Abrahams et al., 2017; Wang et al., 2022; Amidi et al., 2022; van den Berg et al., 2015). Four studies were rated as low-risk incomplete outcome data (Holtdirk et al., 2021; Akkol-Solakoglu and Hevey, 2023; Abrahams et al., 2017; van den Berg et al., 2015) and 10 studies received Intention-To-Treat (ITT) to address missing data (Yu et al., 2022; Gong et al., 2021; Holtdirk et al., 2021; Rosen et al., 2018; Akkol-Solakoglu and Hevey, 2023; Abrahams et al., 2017; Peng et al., 2022; Wang et al., 2022; Amidi et al., 2022; van den Berg et al., 2015) (Figures 2, 3).

### Outcome measurement

The studies included in the present meta-analysis assessed anxiety (*n* = 5), depression (*n* = 6), fatigue (*n* = 4), and QOL (*n* = 7). Anxiety was measured using 7-items Generalized Anxiety Disorder Scale (GAD-7), State Anxiety Inventory Scale (SAI), Self-Rating Anxiety Scale (SAS), and Hospital Anxiety and Depression Scale-Anxiety (HADS-A), higher scores on these scales indicate more anxiety. Depression was assessed using various scales including The Patient Health Questionnaire-9 (PHQ-9), Self-rating depression scale (SDS), Hospital Anxiety and Depression Scale-Depression (HADS-D), and Beck Depression Inventory, Second Edition (BDI-II). Also, higher scores indicate more severe depression. Fatigue was assessed using scales including Brief Fatigue Inventory (BFI), The CIS-Fatigue Severity (CIS), and The Functional Assessment of Chronic Illness Therapy–Fatigue (FACIT-F) the higher the FACIT-F score, the lower the fatigue, and the other two scales were reversed. Furthermore, the QOL was measured using two scales, namely European Organization for Research and Treatment of Cancer Quality of Life Questionnaire Core 30 (EORTC-QLQ) and WHO quality of life questionnaire BREF (WHO QOL BREF). The quality of life scales use a higher score for better quality of life.

### Results of the meta-analysis

#### Anxiety

**Figure 4:**
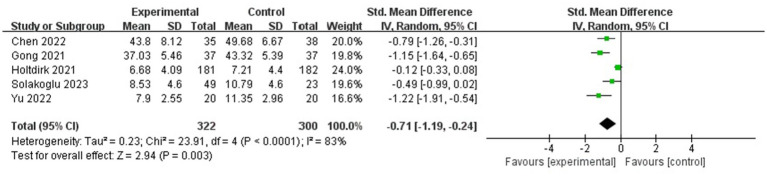
Forest plot for the effect of ICBT on anxiety.

Heterogeneity was found in the five included studies, which involved 622 participants in total (Figure 4) (*p* < 0.05, *I*^2^ = 83%); thus, a random-effects model was selected, and a sensitivity analysis was performed. The sensitivity analysis results showed a statistically significant effect of the intervention (SMD = −0.71, 95% confidence interval [CI]: −1.19, −0.24, *p* < 0.05).

#### Depression

**Figure 5:**
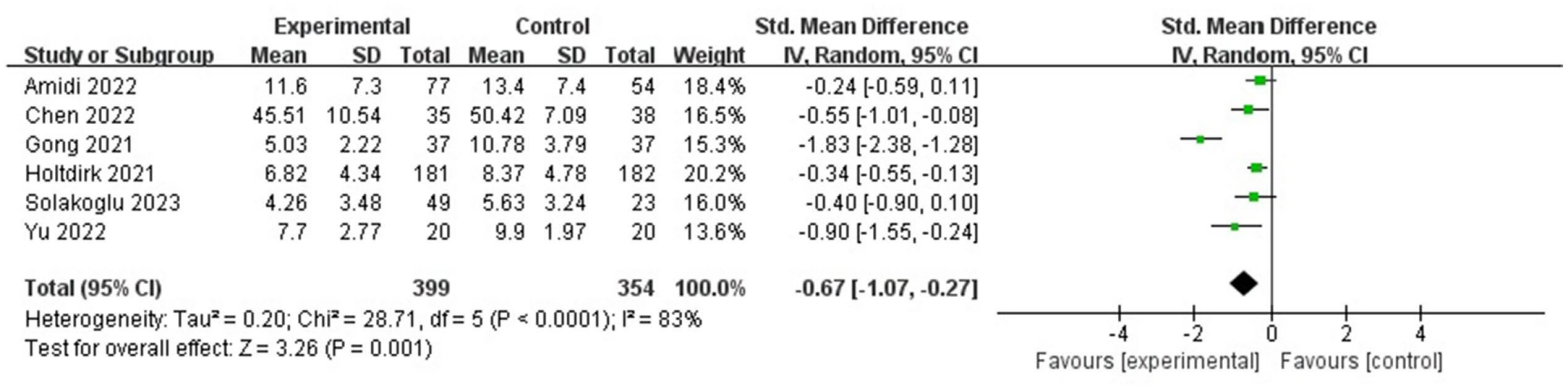
Forest plot for the effect of ICBT on depression.

Heterogeneity was found in the six included studies, which involved 753 participants in total (Figure 5) (*p* < 0.05, *I*^2^ = 83%); thus, a random-effects model was selected, and a sensitivity analysis was performed. The sensitivity analysis results showed a statistically significant effect of the intervention (SMD = −0.67, 95% CI: −1.07, −0.27, *p* < 0.05).

#### Fatigue

**Figure 6:**
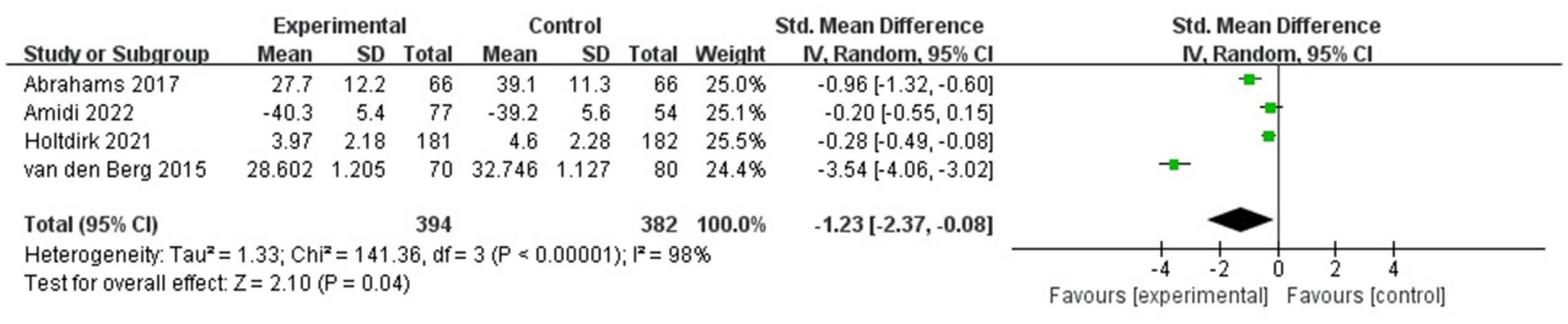
Forest plot for the effect of ICBT on fatigue.

Heterogeneity was found in the five included studies, which involved 622 participants in total (Figure 6) (*p* < 0.05, *I*^2^ = 98%); thus, a random-effects model was selected, and a sensitivity analysis was performed. The sensitivity analysis results showed a statistically significant effect of the intervention (SMD = −1.23, 95% CI: −2.37 − 0.08 *p* < 0.05).

#### QOL

**Figure 7:**
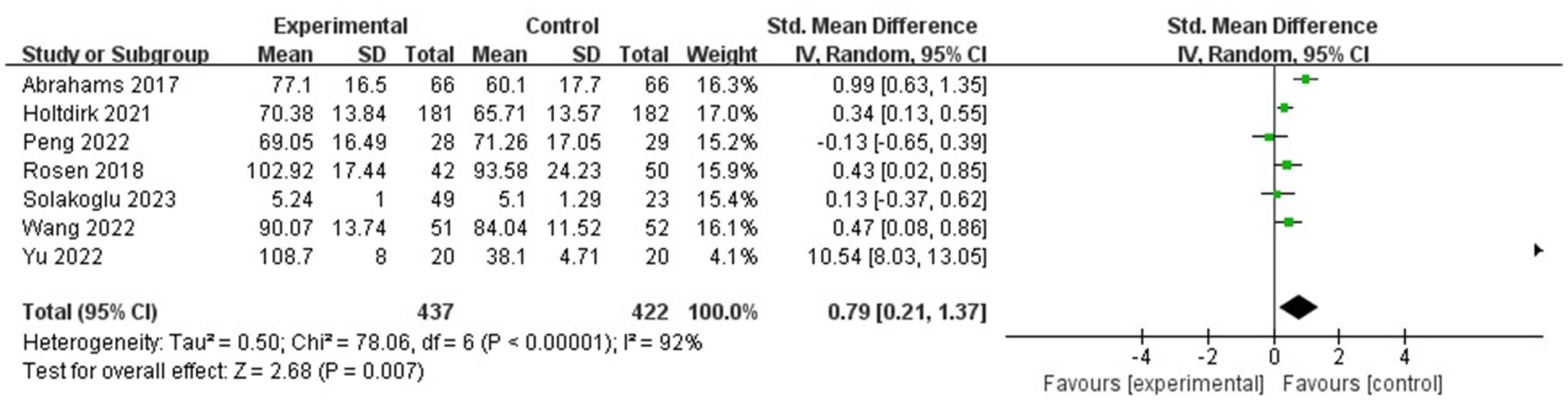
Forest plot for the effect of ICBT on quality of life.

Heterogeneity was found in the seven studies, which involved 859 participants in total (Figure 7) (*p* < 0.05, *I^2^* = 92%); thus, a random-effects model was selected, and a sensitivity analysis was performed. The sensitivity analysis results showed a statistically significant effect of the intervention (SMD = 0.79, 95% CI: 0.21, 1.37, *p* < 0.01), whereas the heterogeneity was high.

## Discussion

Based on the systematic review and meta-analysis, the outcomes showed that ICBT essentially reduced psychological distress in patients with BC. In addition, it was shown that BC patients treated with ICBT had significant improvements in QOL. Additionally, no adverse events related to ICBT were reported in the 11 studies included in this meta-analysis. The present results showed that internet-based interventions were better than general care in ameliorating discomfort in patients with BC, which is in line with Cheung et al.’s results (Cheung et al., 2017). Using internet-based interventions, medical staff could help patients with different types of breast cancer conveniently learn about their treatment, care and home self-care, based on which the patients could make some changes in their cognition, emotions, and behaviors. Furthermore, they were positively eager to learn and master necessary rehabilitation skills and knowledge. Consequently, the patients actively and effectively responded to the treatment. Thus, cognitive intervention and emotional communication were combined in ICBT in order to encourage patients to actively express their feelings. Additionally, in the 11 articles included, patients in the experiment group showed that it was more convenient for them to timely and reasonably cope with psychological problems compared with the control group.

Previous systematic review showed that cognitive-behavioral therapy (CBT) effectively reduced psychological distress and improved the QOL of patients with BC (Ye et al., 2018). A study showed that mindfulness-based cognitive therapy improved the well-being of patients with BC, encompassing their psychological, physical, and spiritual aspects (Park et al., 2020). Although cognitive behavioral therapy (CBT) can improve negative emotions in cancer patients. However, due to the limited number of psychologists, the high cost of treatment, and patients’ feedback that it is difficult to adhere to the full course of treatment, ICBT solves these problems (Li Y. H. et al., 2023; Li Y. et al., 2023; Karageorge et al., 2017; Li and Wang, 2023). A qualitative study also showed high acceptance of ICBT in breast cancer patients (Akkol-Solakoglu and Hevey, 2024). And ICBT was equally effective and potently improved the CBT utilization rate (Zachariae et al., 2018) But this conclusion requires further research before these treatments can have comparable long-term reliability and availability. Additionally, the quality of the included studies and other sociodemographic factors did not affect the observed heterogeneity.

Herein, strict inclusion criteria were followed to minimize heterogeneity in the meta-analysis, which limited analysis to multimodal ICBT compared with comparators in patients with breast cancer. High heterogeneity levels were probably because of the large sample size of one study (Holtdirk et al., 2021). Therefore, we excluded remarkable differences in patients and interventions from the meta-analysis. Additionally, we selected suitable outcomes with low heterogeneity levels; for example, depression and anxiety were the most suitable primary measures for CBT, and the majority of the studies reported data on these variables. We observed distinctive benefits of Internet-based cognitive behavior therapy compared with those of general care or face-to-face cognitive behavioral intervention. Moreover, patients with BC in the intervention groups showed significantly better mental health and QOL than shown by those in the control group; however, whether their fear of cancer recurrence was reduced was not reported. An e-health intervention study on patients with BC with the fear of cancer recurrence showed that CBT markedly reduced the fear of cancer recurrence, and the rate of patient attrition for remote counseling decreased, thus providing insights into future implications (Wagner et al., 2021).

### Clinical implications

The present systematic review provided a detailed overview of the evidence on internet-based cognitive interventions in BC survivors. The results supported that ICBT significantly improved distress in patients with BC. The reduction in distress and a shift toward an internal locus of control in patients resulted in psychological empowerment, which was associated with better psychological health outcomes and QOL during rehabilitation. The present meta-analysis indicated ICBT as an effective intervention for improving QOL and psychological health of patients with cancer. The results showed that diverse behavioral techniques, including behavioral therapy, cognitive therapy, education, and relaxation, were used by these patients to effectively reduce depression and anxiety.

We conducted literature searches using the above search terms, databases, and date restrictions in order to better place our reviews in the context of previous reviews, and we limited our reviews to systematic reviews and meta-analyses published in Chinese and English. Psychotherapy provided by the Internet has been proven to result in a small incremental effect in patients with mental illness (Jonsson et al., 2023). In addition, many systematic reviews and quantitative meta-analyses have found that ICBT are effective for improve anxiety and depression in cancer patients (Yu et al., 2023; Bai, 2023; Liu et al., 2022). Therefore, we believe that the present study is the most accurate estimate of ICBT efficacy under standard conditions for patients with BC compared with control treatments.

### Limitations

Nevertheless, the review has some limitations to address. First, high heterogeneity levels were observed in the included studies owing to the diversity of the country of origin, target population, intervention protocols (intensity, frequency, and duration), measurement tools, and measurement time points. Because the concept of psychosocial interventions based on telemedicine is relatively new, we screened a limited number of studies for further adequate subgroup analysis. Second, inevitably, the included studies used self-reported structural measures that resulted in social expectation bias and ecological fallacy, which were attributed to the comparison of analytical methods and relationships at the study level rather than by examination at the individual objective level. Third, concerning telemedicine interventions, high training and development costs should be considered, which is an important factor in guiding populations to make health-related choices. Fourth, only Chinese and English studies were selected, which resulted in language selection bias.

## Conclusion

The present meta-analysis shows ICBT as a reasonably effective treatment for psychological distress in patients with BC and reports clinically meaningful results. These findings suggest that ICBT can be used as an effective intervention for BC survivors and patients when necessary. However, there are some challenges with treatment over the Internet, such as the inability to determine the long-term effects of the intervention and the impact of an individual’s depression severity on the effectiveness of the intervention. Future research is needed to optimize specific interventions for ICBTs.

## Data Availability

The original contributions presented in the study are included in the article/supplementary material, further inquiries can be directed to the corresponding author.
